# Smoking is associated with a higher risk of unplanned medical visits among adult patients with diabetes, using retrospective electronic medical record data from 2014 to 2016

**DOI:** 10.1186/s12913-020-05277-4

**Published:** 2020-05-06

**Authors:** Arielle Selya, Eric L. Johnson, Tess L. Weber, Jaymi Russo, Cheryl Stansbury, Drake Anshutz, Emily Griese, Benson Hsu

**Affiliations:** 1 Department of Population Health, University of North Dakota School of Medicine & Health Sciences, Grand Forks, ND USA; 2 Behavioral Sciences Group, Sanford Research, 2301 East 60th Street North, Sioux Falls, SD 57104 USA; 3 Department of Pediatrics, University of South Dakota Sanford School of Medicine, Sioux Falls, SD USA; 4 Department of Family & Community Medicine, University of North Dakota School of Medicine & Health Sciences, Grand Forks, ND USA; 5 The Miller Pediatric Critical Care Unit, Sanford Children’s Hospital, Sioux Falls, SD USA

**Keywords:** Smoking, Tobacco, Diabetes, Unplanned medical visits

## Abstract

**Background:**

Smoking exacerbates the complications of diabetes, but little is known about whether patients with diabetes who smoke have more unplanned medical visits than those who do not smoke. This study examines the association between smoking status and unplanned medical visits among patients with diabetes.

**Methods:**

Data were drawn from electronic medical records (EMR’s) from a large healthcare provider in the Northern Plains region of the US, from adult (≥18 years old) patients with type 1 or type 2 diabetes who received care at least once during 2014–16 (*N* = 62,149). The association between smoking status (current, former, or never smoker) and having ≥1 unplanned visit (comprised of emergency department visits, hospitalizations, hospital observations, and urgent care) was examined after adjusting for age, race/ethnicity, and body mass index (BMI). The top ten most common diagnoses for unplanned visits were examined by smoking status.

**Results:**

Both current and former smoking were associated with an approximately 1.2-fold increase in the odds of having at least one unplanned medical visit in the 3-year period (OR = 1.22, 95% CI = 1.16–129; OR = 1.23, 95% CI = 1.19–1.28, respectively), relative to never-smokers. Most common diagnoses for all patients were pain-related. However, diagnoses related to musculoskeletal system and connective tissue disorders were more common among smokers. Smoking is associated with a higher rate of unplanned medical visits among patients with diabetes in this regional healthcare system.

**Conclusions:**

Results from this study reveal higher rates of unplanned visits among smokers and former smokers, as well as increased frequencies of unplanned medical visits among current smokers.

## Background

In 2018, approximately 34.2 million persons in the United States or 10.5% of the population had diabetes, and about 1.5 million new cases are diagnosed every year among people 18 and over [1]. Both macrovascular and microvascular complications are more common in this population than in the general population. Infections including soft tissue, respiratory tract, and urinary tract infections are also more common among persons with diabetes [2]. As a result, Americans with diabetes in 2017 spent approximately $16,700 annually in health care costs, 2.3 times higher than those without diabetes [3]. Total costs of diabetes in 2017 were $327 billion annually, of which $237 billion were in direct medical costs [3].

Cigarette smoking is a well-established risk factor for complications of diabetes. Smoking decreases glycemic control [4], increases risk of infection [5, 6], and amplifies an already elevated risk of cardiovascular events [7, 8]. Patients with diabetes are more likely to have unplanned medical visits [9, 10], but less is known about the impact of smoking on health care utilization and costs among patients with diabetes. A study following 206 patients with diabetic foot ulcers found 17% of patients had a 30-day unplanned readmission, noting current smoking and hypertension as independent predictors of readmission [11]. However, little research has looked at the association between smoking and unplanned medical visits more generally among this high-risk population.

Another issue adding to an already complex topic concerns the varied smoking regulatory environments individual states enact. The Congressional Budget Office [12] found that a 10% cigarette price increase will result in a 3–5% decrease in consumption, helping to improve public health. As of 2019, the average state cigarette tax is $1.70 per pack, with Washington DC having the highest tax ($4.50/pack) and Missouri having the lowest ($0.17/pack) [13]. Varying regulatory environments have the potential to impact smoking behaviors among individuals living in those states, an important variable to consider when examining smoking behaviors across state lines.

The primary objective of this retrospective study was to examine the relationship between smoking status and unplanned medical visits among patients with diabetes through electronic medical record (EMR) data from Sanford Health, a regional healthcare provider with a large patient base in Minnesota, North Dakota, and South Dakota. A sub-aim was to examine these relationships across different states with varied regulatory environments. The states represented in the study range broadly in their cigarette excise tax rates with North Dakota having the lowest taxes at $0.44 per pack (Rank 48th) [14], Minnesota having one of the highest taxes at $3.04 (Rank 8th) [15], and South Dakota in the middle ($1.53) with its ranking of 28th [13, 16].

## Methods

### Sample

The dataset was custom-made by Sanford Health and Sanford Research’s Data Collaborative, and includes electronic medical records, claims data, and event level data. All data are de-identified according to the Health Insurance Portability and Accountability Act HIPAA de-identification method Safe Harbor § 164.514(b)(2). The data set covers records from 2014 to 2016 (a three-year period) and includes *N* = 1,143,028 patients who received care at least once within a Sanford facility during this period. Sanford Health is a not-for-profit rural healthcare system that primarily serves South Dakota, North Dakota, Northern & Southwest Minnesota, Northwest Iowa, and parts of Nebraska, and includes roughly 44 hospitals, 1382 physicians and 9703 nurses delivering care in more than 80 specialty areas. Sanford uses Epic software for EMRs, and this custom dataset was pulled by Sanford’s Enterprise Data & Analytics (EDA) team. The beginning of the time period for these data (starting 2014) coincides with the beginning of quality-controlled data in this system.

For this study, those under 18 years of age were excluded (*N* = 267,860), as were those whose most recent residential zip code was outside of Minnesota (MN), North Dakota (ND), or South Dakota (SD) (*N* = 65,980) due to low sample sizes in other states. Records with missing data on smoking status were also excluded (*N* = 34,742). Finally, the sample was restricted to those with a diagnosis of diabetes (ICD-10 codes E10.xx and E11.xx), for a final sample size of *N* = 62,149.

### Measures

Unplanned medical visits was derived from four separate variables for the numbers of 1) emergency department visits, 2) hospitalizations, 3) hospital observations, and 4) urgent care visits over the 3-year period. Two versions of the combined variable were created: a numeric variable representing the sum of all types of unplanned visits, and a binary variable indicating any (1+) or no (0) unplanned visits over the 3-year period.

Smoking status was obtained from EMR’s and was re-categorized as current smoking (collapsing the original levels of current every day smoker, current some day smoker, heavy tobacco smoker, light tobacco smoker, and smoker with current status unknown) vs. former smoking vs. never-smoking (collapsing the original levels of never smoker and passive smoke exposure). Since smoking status can change over time, the most recent smoking status was used.

Body mass index (BMI) was obtained from EMR’s. Extreme values of BMI < 15 or > 60 were assumed to be errors and were set to missing.

Race/ethnicity was collected from EMR’s in multiple variables for each race/ethnic group endorsed by the patient. For the current study, due to the predominantly white/Caucasian sample (87.9%), this was dichotomized as white (endorsed only white/Caucasian), vs. non-white (endorsed black/African American, American Indian/Alaska, Hispanic/Latino, Asian, Native Hawaiian/Pacific Islander, and/or multiracial).

Age and sex were also obtained from EMR’s

Primary diagnoses for unplanned visits were available as ICD-10 codes for EMR’s from emergency department visits and urgent care visits over the 3-year period. ICD-codes were stripped to their prefix (e.g. “E11” instead of “E11.3”) to examine generally which conditions lead to unplanned medical visits, rather than focusing on highly specific diagnoses.

### Analyses

First, unadjusted relationships between smoking status and unplanned medical visits were examined using chi-square tests (for any vs. no unplanned visits) and the non-parametric Kruskal-Wallis rank sum test (for the number of unplanned medical visits) due to the non-normality of the outcome variable.

Second, logistic regression was used to examine the odds of having an unplanned medical visit over the 3-year period as a function of smoking status while adjusting for age, sex, race/ethnicity, BMI, and state (MN, ND, and SD). We made an intentional decision not to adjust for additional behavioral and medical covariates, for the purpose of conducting a pragmatic study which is more generalizable to real-world heterogeneous clinical populations (see Discussion).

Missing data analyses were not performed because the proportion of missing data on smoking status was only 4.3% of the dataset, and is therefore unlikely to meaningfully impact the findings.

Follow-up analyses were performed separately by state (MN, ND, SD) in order to account for the different tobacco regulatory environments.

Finally, the 10 most common primary diagnoses from unplanned visits were tabulated within each smoking group for purposes of comparison.

Additional analyses that were run but not presented in the final manuscript included a single model that aggregated across states, subgroup analyses within each state, and a model that included numeric tax rates per pack of cigarettes instead of state. All of these analyses yielded nearly identical results to the ones presented here.

## Results

Overall, 14.6% of the sample were current smokers, 39.5% were former smokers, and 45.9% had never smoked. Current smoking rates were similar across states, with a slightly higher prevalence in Minnesota (15.5%) than North Dakota (14.0%) or South Dakota (13.9%), and slightly more former smokers in Minnesota (41.6%) than in North Dakota (38.4%) or South Dakota (38.0%).

Descriptive statistics of patients with diabetes broken down by smoking status and state are shown in Table 1. Among the pooled sample, current smokers were most likely to have at least one unplanned medical visit over the 3-year period (59.1%), followed by former smokers (56.6%), with never-smokers having the lowest percentage (51.9%). Similarly, among patients with at least one unplanned medical visit over the 3-year period, current smokers (median: 3; interquartile range (IQR): 2–7) and former smokers (median: 3, IQR = 1–6) had a greater *number* of unplanned visits than never-smokers (median: 2, IQR: 1–5). Additionally, never-smokers were more likely to be white (91.1%) and less likely to be male (44.6%). However, trends in BMI and sex did not show a consistent pattern across smoking categories: former smokers had the highest BMI (median: 32.4, interquartile range (IQR): 28.3–37.3) and the highest percentage of males (62.1%). All of these same trends held within each state (MN, ND, and SD). Current smokers tended to be younger (median: 56 years, IQR: 54–77) than never-smokers (median: 69 years, IQR = 60–77), who in turn tended to be younger than former smokers (median: 69 years, IQR = 60–77).

**Table 1:** Descriptive statistics by smoking status

	State	Never-Smoker *N* = 28,543	Former Smoker *N* = 24,550	Current Smoker *N* = 9056	*p*
Any unplanned visits	MN	48.1% (4834)	53.7% (5223)	58.2% (2118)	<.001
	ND	55.7% (5586)	59.9% (4854)	59.8% (1770)	<.001
	SD	51.8% (4388)	57.0% (3823)	59.6% (1463)	<.001
	Total	51.9% (14808)	56.6% (13900)	59.1% (5351)	<.001
Number of unplanned visits	MN	2 (1–5)	3 (1–6)	3 (2–7)	<.001
	ND	2 (1–5)	3 (1–6)	3 (2–7)	<.001
	SD	2 (1–4)	3 (1–5)	3 (1–6)	<.001
	Total	2 (1–5)	3 (1–6)	3 (2–7)	<.001
BMI	MN	32.1 (28.0–37.1)	32.5 (28.4–37.3)	31.6 (27.1–36.8)	<.001
	ND	32.1 (28.1–37.2)	32.4 (28.3–37.3)	31.5 (27.2–36.6)	<.001
	SD	32.0 (27.8–37.3)	32.3 (28.2–37.2)	31.2 (26.8–36.5)	<.001
	Total	32.1 (28.0–37.2)	32.4 (28.3–37.3)	31.5 (27.1–36.7)	<.001
Non-Hispanic white	MN	91.0% (9017)	88.6% (8510)	69.8% (2507)	<.001
	ND	90.4% (8925)	90.1% (7206)	81.3% (2375)	<.001
	SD	92.0%(7753)	88.2% (5883)	74.2% (1810)	<.001
	Total	91.1% (25695)	89.0 (21599)	74.8 (6692)	<.001
Male	MN	43.6% (4379)	62.8% (6109)	53.2% (1938)	<.001
	ND	45.5% (4558)	62.7% (5085)	55.7% (1649)	<.001
	SD	44.9% (3805)	60.4% (4049)	52.3% (1282)	<.001
	Total	44.6% (12742)	62.1% (15243)	53.8% (4869)	<.001
Age	MN	67 (56–78)	70 (61–78)	57 (46–65)	<.001
	ND	64 (53–76)	68 (59–77)	56 (46–64)	<.001
	SD	64 (53–76)	68 (59–77)	56 (46–64)	<.001
	Total	65 (54–77)	69 (60–77)	56 (46–65)	<.001
Alcohol Use	MN	34.8% (3382)	41.8% (3971)	39.7% (1396)	<.001
	ND	38.4% (3735)	42.7% (3382)	45.5% (1311)	<.001
	SD	37.5% (3139)	41.1% (2730)	43.8% (1060)	<.001
	Total	36.8% (10,256)	41.9% (10,083)	42.7% (3767)	<.001

Note: Categorical variables are presented as % (*N*) and numeric variables are presented as median (interquartile range). Significant differences by smoking status were tested with chi-square tests for categorical variables and Kruskal-Wallis rank sum tests for numeric variables. *MN* Minnesota. *ND* North Dakota. *SD* South Dakota. *BMI* body mass index

The logistic regression (Table 2) showed that among patients with diabetes, both current and former smokers were more likely to have had at least 1 unplanned medical visit in the 3-year period (current smokers: OR = 1.24, 95% confidence interval (CI) = 1.20–1.29; former smokers: OR = 1.23, CI = 1.17–1.29) relative to never-smokers, after adjusting for white race, sex, and BMI. Follow-up analyses failed to find a significant difference between the odds of at least 1 unplanned medical visit between former smokers and current smokers (*p* = .654). Analyses by state (data not shown) were highly similar to the aggregate results shown in Table 2: both former and current smokers were significantly more likely to have had an unplanned medical visit in the 3-year period, relative to nonsmokers. Additionally, the odds of unplanned medical visits related to smoking status did not differ across state (MN, ND, or SD), judging by overlapping 95% confidence intervals; nor did ex-smokers differ from current smokers in terms of their odds of having an unplanned medical visit.

**Table 2:** Logistic regression results of unplanned medical visits

		OR	95% CI	*p*
Tobacco use	Never-smoker	(Ref)	(Ref)	(Ref)
	Former smoker	1.24	(1.20–1.29)	<.001
	Current smoker	1.23	(1.17–1.29)	<.001
BMI		1.00	(1.00–1.00)	.090
Race	White	(Ref)	(Ref)	(Ref)
	Non-white	1.75	(1.65–1.85)	<.001
Sex	Male	(Ref)	(Ref)	(Ref)
	Female	1.20		<.001
State	Minnesota	(Ref)	(Ref)	(Ref)
	North Dakota	1.29	(1.24–1.34)	<.001
	South Dakota	1.06	(1.02–1.10)	.007

Note: *OR* odds ratio. *CI* confidence interval. *BMI* body mass index

Table 3 shows the most common diagnoses from emergency department and urgent care visits during the 3-year period. Smokers and nonsmokers had a highly similar profile of diagnoses: the rank order of the five most common diagnoses was the same across the two groups, consisting mainly of pain-related diagnoses. However, most of these diagnoses were more common among smokers. Additionally, codes related to musculoskeletal system and connective tissue diagnoses tended to have a higher percentage among smokers in comparison to nonsmokers. Conversely, “pain in throat and chest” and “cough” were more frequent among nonsmokers than among current or former smokers.

**Table 3:** Most common diagnoses from emergency room and urgent care visits, by smoking status

Smokers with Diabetes		Nonsmokers with Diabetes	
Diagnoses	Frequency	Diagnoses	Frequency
R10.xx: Abdominal and pelvic pain	6.6% (*N* = 4109)	R10.xx: Abdominal and pelvic pain	5.4% (*N* = 8856)
M54.xx: Dorsalgia	6.1% (*N* = 3750)	M54.xx: Dorsalgia	4.3% (*N* = 7099)
R07.xx: Pain in throat and chest	3.6% (*N* = 2252)	R07.xx: Pain in throat and chest	4.3% (*N* = 6971)
M25.xx: Other joint disorder, not elsewhere classified	3.4% (*N* = 2114)	M25.xx: Other joint disorder, not elsewhere classified	3.2% (*N* = 5175)
M79.xx: Other and unspecified tissue disorders	3.4% (*N* = 2085)	M79.xx: Other and unspecified tissue disorders	3.1% (*N* = 5092)
L03.xx: Cellulitis and acute lymphangitis	2.3% (*N* = 1453)	R05.xx: Cough	2.8% (*N* = 4547)
E11.xx: Type II Diabetes Mellitus	2.1% (*N* = 1314)	L03.xx: Cellulitis and acute lymphangitis	2.2% (*N* = 3526)
R05.xx: Cough	2.1% (*N* = 1298)	J40.xx: Bronchitis, not specified as acute or chronic	2.1% (*N* = 3422)
J40.xx: Bronchitis, not specified as acute or chronic	2.0% (*N* = 1265)	J02.xx: Acute pharyngitis	2.0% (*N* = 3222)
G43.xx: Migraine	1.9% (*N* = 1197)	R51.xx: Headache	1.8% (*N* = 3140)

## Discussion

This paper utilizes regional EMR data to examine the relationship between smoking status and unplanned medical visits among patients with diabetes in ND, MN, and SD over a 3-year period. Results show that current and former smokers were more likely to have unplanned medical visits, which include emergency department, hospitalization, hospital observation, and urgent care visits. Further, among the diabetic patients who did have unplanned medical visits, current smokers had the greatest frequency of unplanned visits. The diagnoses from the unplanned emergency department and urgent care visits among smokers and non-smokers were similar and often related to pain (e.g. abdominal pain which may be due to uncontrolled glucose); however, smokers had a slightly higher proportion of visits related to connective tissue or musculoskeletal disorders compared to non-smokers.

Patients with diabetes generally have higher healthcare utilization compared to those without diabetes, including clinic visits, outpatient departments, and emergency departments [11, 17, 18]. The 2011 National Health Interview Survey revealed that 30% of diabetic patients had at least one emergency department visit within the last year, compared to only 20% of the general population [17]. While increased healthcare utilization is evident among diabetics, less research has focused on how smoking among diabetics is associated with healthcare utilization, specifically on unplanned medical visits. Our findings are novel in showing that smokers with diabetes are more likely to have unplanned medical visits than non-smokers with diabetes, delineating between former and current smokers as well as differences across states and their associated smoking regulations. Prior studies have suggested increased health resource utilization among diabetics who are also smokers [9], however these findings did not delineate among current and former smokers and the association specifically with unplanned medical visits [10].

Our findings that smokers with diabetes had higher rates of musculoskeletal and connective tissue disorders is consistent with prior literature [19, 20]. Smoking has been found to heighten these complications given its effect on bone mineral density and adverse effects on joints [21, 22]. There has been early findings suggesting that smoking cessation can help regenerate lost bone and joint health, but reversing these complications takes extended time [21], which may explain the lack of statistical difference between current and former smokers in the present findings. Unfortunately, given nicotine’s therapeutic effects for chronic pain in smokers, smoking cessation may not be successful when pain persists and smoking provides a level of short-term alleviation [23]. Intervention efforts that focus on alleviation of pain associated with musculoskeletal and connective tissue disorders through other modalities may assist in removing a barrier to successful smoking cessation efforts.

Respiratory illnesses, in general, are common among diabetic patients [24]. Irregular insulin levels may influence known respiratory illness given the influx of blood glucose, harming vital tissue function [25]. Smokers, however, are less likely to seek treatment for respiratory illnesses such as cough than nonsmokers [26]. Complications in this regard for smokers lend to the notion that smokers seek tobacco as a potential treatment form in place of urgent care [27]. This could explain, in part, why the current study found that fewer current or former smokers reported respiratory complaints as compared to non-smokers.

Novel findings suggested differences by state in the prevalence of unplanned medical visits, with patients in Minnesota having the lowest rates of unplanned visits, followed by South Dakota, and finally North Dakota. This mirrors the tax rates associated with tobacco in each state, which is highest in Minnesota and lowest in North Dakota. Surprisingly, however, the smoking *prevalence* did not show the expected trends, with slightly more smokers in Minnesota ($3.04/pack) compared to North ($0.44/pack) and South Dakota ($1.53/pack). Nevertheless, there were no differences by state in the *association* between smoking and unplanned medical visits, meaning that smoking poses the same risk for unplanned medical visits despite differences in prevalence. In other words, once patients started smoking, they had similar risk for unplanned medical visits regardless of the tax environment. If these findings reflect a causal relationship between smoking and unplanned medical visits, this study highlights the importance of primary prevention of smoking in primary care settings, and the corresponding high-cost healthcare utilization.

### Limitations

The observational nature of the data used in this study may be considered a limitation given no causal relationship between smoking and unplanned medical visits among diabetic patients can be inferred. Additionally, the findings related to most common diagnoses may not be representative, as diagnosis data were only available from urgent care visits and emergency department visits.

Additionally, smoking status was available only from the most recent visit; this allows for the possibility of reverse causality (unplanned medical visits could conceivably have caused smoking). Ideally, the earliest value of smoking status would have been used, to be consistent with the assumed temporal relationship in this study.

We controlled for only demographic characteristics in this study, and intentionally excluded other potential confounders is related to behavioral health (e.g. physical activity, alcohol use) and medical outcomes (e.g. complications or severity of diabetes). The implications of this decision are that the relationship may not be entirely attributable to smoking. Nevertheless, our rationale was to conduct a pragmatic study, which is more generalizable to real-world heterogeneous clinical populations, when compared with strongly controlled studies [28, 29](REF). For example, results from drug trials in randomized controlled trials (RCTs) often fail when rolled out to the general population, due to the RCT population having better health and compliance [30] (REF). In this context, since smoking is highly correlated with other behavioral and medical health factors, we decided a pragmatic approach was appropriate to capture the real-world outcomes associated with smoking in a heterogeneous patient population. This study examines data from ND, MN and SD, and may be limited in its generalizability other populations. The sample is however relatively proportional to the patient population of the Northern Plains.

Future research is needed to overcome some of these limitations and advance our findings. For example, longitudinal data could be used with a fixed effects panel analysis to examine the temporal relationship between smoking and unplanned visits. Additionally, this research question can be expanded to other groups, for example to compare whether smoking has a stronger relationship with unplanned medical visits among patients with vs. without diabetes.

### Strengths

It is well known that diabetic patients may have unplanned medical visits for other diabetes-related complications [31, 32] and few studies have shown higher rates of tobacco use among emergency department visits [32, 33]; however, exploring the relationship between diabetes and smoking in regards to unplanned visits is a novel concept. This study utilizes a very large sample size representative of the patient population in the Northern Plains, an important and often understudied region in the US.

## Conclusions

Smoking is associated with more frequent unplanned medical visits among patients with diabetes. While there is little literature examining healthcare utilization among diabetic patients as a function of smoking status, results from this study reveal higher rates of unplanned visits among smokers and former smokers, as well as increased frequencies of unplanned medical visits among current smokers. These findings add to the pervasive and well-established health risks of smoking [34]. Healthcare delivery settings represent a valuable opportunity for smoking cessation referrals, e.g. to existing state quit lines, which may especially benefit patients with diabetes in reducing costly healthcare utilization.

## Data Availability

The de-identified datasets analyzed during the current study are available from the corresponding author on reasonable request.

## Abbreviations

EMR: Electronic medical record
IQR: Inter-quartile range
OR: Odds ratio
CI: Confidence interval
BMI: Body mass index
MN: Minnesota
ND: North Dakota
SD: South Dakota
